# How much does a disrupted mitochondrial network influence neuronal dysfunction?

**DOI:** 10.15252/emmm.201809899

**Published:** 2018-12-14

**Authors:** Zofia MA Chrzanowska‐Lightowlers, Robert N Lightowlers

**Affiliations:** ^1^ Wellcome Centre for Mitochondrial Research Institute of Neuroscience Medical School Newcastle University Newcastle upon Tyne UK; ^2^ Wellcome Centre for Mitochondrial Research Institute for Cell and Molecular Biosciences Medical School Newcastle University Newcastle upon Tyne UK

**Keywords:** Genetics, Gene Therapy & Genetic Disease, Neuroscience

## Abstract

Mitochondria are organelles that are present in all nucleated cells in the body. They have manifold functions but famously generate ATP efficiently through the process of oxidative phosphorylation. This ensures all tissues have an adequate energy supply and underlines the need for a fully functional mitochondrial network. Since mitochondrial biogenesis and maintenance require components from two genetic sources, mitochondrial diseases can result from mutations in either the nuclear or the mitochondrial genome (mtDNA). Enigmatically, mitochondrial disease can affect individuals at any age and in any tissue (Lightowlers *et al*, 2015). For a subset of mutations, the genotype can be ascribed to a clinical phenotype and a number of mutations are associated with remarkable tissue selectivity (Boczonadi *et al*, 2018). However, the gene expression pathways governing this tissue‐specific presentation are far from clear. In this issue of *EMBO Molecular Medicine*, Sprenger *et al* (2019) use mouse models to investigate the consequences of deleting a mitochondrial protease, YME1L, in neuronal/glial precursors. The loss causes multiple defects at both cell and tissue level, including a marked fragmentation of the mitochondrial network. Tandem depletion of a second mitochondrial protease, Oma1, successfully restored the mitochondrial connectivity, but did not rescue the ocular defects and caused an earlier onset of neurological dysfunction. Thus, in addition to other findings, the authors conclude that a fragmented mitochondrial network contributes less to the disease phenotype than the disruption of mitochondrial proteostasis.

Mitochondria perform a number of key functions within the cell but are probably most frequently associated with the production of ATP. To assemble much of the chain of enzyme complexes that perform oxidative phosphorylation (OXPHOS) requires coordinated expression and assembly of gene products from both the nuclear and the mitochondrial genome. The catalogue of diseases associated with mitochondrial dysfunction continues to increase, and in many cases, the ubiquity of exome or whole genome sequencing has enabled the identification of the genetic mutations responsible for these pathogenic defects (Taylor *et al*, 2014). At present, there is no cure for these diseases and treatment, where possible, is currently limited to ameliorating the symptoms of the common presentations, rather than addressing the cause. Work on preventing the transmission of mtDNA mutations is ongoing (Herbert & Turnbull, 2018), but this will not address the problems caused by nuclear‐encoded defects. To develop potential therapies for these conditions, which can be varied in presentation, we need to increase our knowledge of the specific consequences of the mutations in a whole organism.

Amongst the different tissue phenotypes, neuronal dysfunction or degradation are common features of mitochondrial disease. However, determining the molecular pathogenesis is challenging. The use of mouse models to address such questions can be very informative. This approach has been adopted by the Langer laboratory to investigate the molecular consequences of mutations in the mitochondrial *i‐*AAA protease, YME1L. The latter is an enzyme that is responsible for the homeostasis both of mitochondrial proteins and phospholipids. It does so mostly by removing proteins that are damaged or have not been correctly assembled into functional complexes and by degrading short‐lived lipid transfer proteins (Coenen *et al*, 2005; Stiburek *et al*, 2012). Essentially, this prevents the organelle becoming clogged up with dysfunctional debris that would impede mitochondrial function. Additionally, one of the YME1L targets is a dynamin‐like GTPase, OPA1, which is crucial for maintenance of mitochondrial dynamics through its involvement in mitochondrial fusion and cristae formation. Pathogenic mutations in *YME1L* have been identified, and patients harbouring homozygous recessive mutations frequently present with a neuromuscular disorder including movement disturbances and optic atrophy (Hartmann *et al*, 2016). At a cellular level, the mitochondrial network becomes highly fragmented in the affected tissues. This is explained by the stress‐activated cleavage of the long isoform of Opa1 (L‐Opa1) by a different protease, Oma1, in the *Yme1l* knockout mouse model. This longer isoform is essential for promoting the fusion of the inner mitochondrial membrane (Ban *et al*, 2017) and its absence results in a loss of fusion. Interestingly, although fragmented mitochondrial networks can be associated with defects in oxidative phosphorylation, loss of Yme1l in mice caused disrupted mitochondrial morphology and neurological defects independently of any significant deficits in oxidative phosphorylation.

What is the relationship between this change in mitochondrial morphology, eye defects and axonal degeneration with age? Sprenger *et al* have used two knockout mouse models to address this question. One mouse line has been generated in which *Yme1l* is specifically knocked out in neuronal and glial cell precursors. The second line eliminates both YME1L and the IM peptidase, OMA1, which can cleave OPA1 but is usually only activated under stress conditions (Zhang *et al*, 2014). Sprenger *et al* have characterised the phenotypes arising from both the individual or combined neuronal‐specific knockout mice (Fig 1). In humans, mutations in *YME1L* cause ocular dysfunction and movement disturbances. The mouse model eliminating *Yme1l* alone displayed a comparable phenotype with ocular defects including retinal inflammation, cataracts and microphthalmia, a developmental condition where the eyes are abnormally small. This condition is generally associated with increased caspase 9‐mediated cell death but in the absence of YME1L there was no apparent stimulation of this pathway, suggesting a different cause for the microphthalmia. Investigating the temporal nature of the neurological impairment identified that the eye‐related defects (microphthalmia, cataracts, retinal disorganisation) occurred early on, whilst the axonal and locomotor degeneration only developed later, and were not the consequence of generalised brain atrophy. The tandem knockout of both YME1L and OMA1 restored the dynamic mitochondrial network, but despite this restoration of L‐OPA1, not only did the combination of eye defects persist but both the neuroinflammation and axonal degeneration became more severe, with an earlier onset. The combination of models used in these investigations brings us a little closer to understanding pathogenic mechanisms and that disruption of mitochondrial proteostasis alone can be a driver for disease.

**Figure 1:**
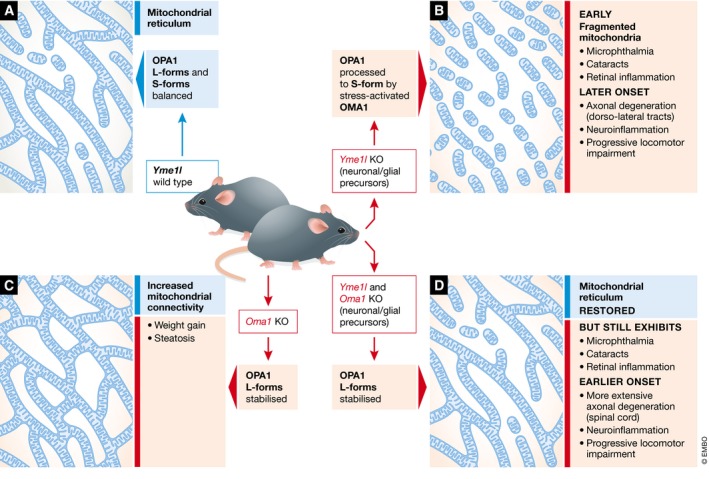
A connected mitochondrial network is not sufficient to overcome the physiological defects arising from a loss of YME1L (A) YME1L activity in wild‐type mice maintains a balance of OPA1 forms to retain a dynamic mitochondrial reticulum. (B) Loss of YME1L from neuronal and glial precursors results in a fragmented mitochondrial network, eye defects and late onset of neuroinflammation, with degeneration of dorso‐lateral tract‐specific axons leading to locomotor impairment. (C) Loss of OMA1 results in stabilisation of L‐OPA1 with increased connectivity of mitochondrial tubules, with mild lipid‐related symptoms under control conditions (Quiros *et al*, 2012). (D) When both of the proteases responsible for processing of OPA1 are knocked out, the reticular nature of the mitochondrial network is restored but the defects associated with loss of YME1L alone are retained or even exacerbated.

## Conflict of interest

The authors declare that they have no conflict of interest.
